# Agarwood—The Fragrant Molecules of a Wounded Tree

**DOI:** 10.3390/molecules27113386

**Published:** 2022-05-24

**Authors:** Pooja Shivanand, Nurul Fadhila Arbie, Sarayu Krishnamoorthy, Norhayati Ahmad

**Affiliations:** 1 Environmental and Life Sciences Program, Faculty of Science, Universiti Brunei Darussalam, Jalan Tungku Link, Bandar Seri Begawan BE1410, Brunei; 18b2114@ubd.edu.bn (N.F.A.); norhayati.ahmad@ubd.edu.bn (N.A.); 2 Department of Civil Engineering, Environmental Water Resources Engineering Division, Indian Institute of Technology Madras, Chennai 600 036, India; sarayuk1528@gmail.com; 3 Institute for Biodiversity and Environmental Research, Universiti Brunei Darussalam, Jalan Tunku Link, Bandar Seri Begawan BE1410, Brunei

**Keywords:** agarwood, *Aquilaria*, artificial induction, bioactive compounds, chromones, terpenoids

## Abstract

Agarwood, popularly known as oudh or gaharu, is a fragrant resinous wood of high commercial value, traded worldwide and primarily used for its distinctive fragrance in incense, perfumes, and medicine. This fragrant wood is created when Aquilaria trees are wounded and infected by fungi, producing resin as a defense mechanism. The depletion of natural agarwood caused by overharvesting amidst increasing demand has caused this fragrant defensive resin of endangered *Aquilaria* to become a rare and valuable commodity. Given that instances of natural infection are quite low, artificial induction, including biological inoculation, is being conducted to induce agarwood formation. A long-term investigation could unravel insights contributing toward *Aquilaria* being sustainably cultivated. This review will look at the different methods of induction, including physical, chemical, and biological, and compare the production, yield, and quality of such treatments with naturally formed agarwood. Pharmaceutical properties and medicinal benefits of fragrance-associated compounds such as chromones and terpenoids are also discussed.

## 1. Introduction

Agarwood, eaglewood, oud, aloeswood, and *gaharu* are some of the names of the highly valuable fragrant heartwood used for nontimber purposes and produced by the *Aquilaria* species, which belongs to the *Thymelaeaceae* family. These “woods of the Gods” have been used and traded for thousands of years for making perfume, which continue to be used in cultural and religious ceremonies, and for the production of incense sticks and fragrance products. It is also used in traditional medicine and has been recorded in the Ayurvedic medicinal text, the *Susruta Samhita* of prehistoric times, and in the *Sahih Muslim* that dates to the 8th century [1]. Agarwood oil is a desirable product and is traded worldwide, with high demand in Japan, China, the United Arab Emirates, and Saudi Arabia. It has a high demand in the Middle East because the oil symbolizes wealth, culture, and hospitality [2]. The value of agarwood depends on its quality, geographical location, and uses in a culture. Agarwood chips can cost from £20 per kilo up to £6000 per kilo depending on the amount of resin in the chips, while the agarwood oil can cost as much as £20,000 depending on the purity [3].

There are 21 *Aquilaria* species, with 9 agarwood (resin)-producing species [4,5], and they are found in India, New Guinea, Hainan Island in China, and in Southeast Asian countries [6]. Akter [7] stated that the formation of agarwood is associated with wounding of the tree and fungal invasion, causing the tree to produce a fragrant resin high in volatile compounds that helps suppress the growth of fungi. Increased damage would eventually cause more resin to be produced.

### Aquilaria

Due to its importance in culture, religion, and value, agarwood is highly sought after, and the trees are often overexploited and traded illegally, which has placed it on the list of endangered species in Appendix II of the Convention on International Trade in Endangered Species of Wild Flora and Fauna (CITES) in 1995 [1]. Furthermore, Chakrabarty et al. [8] reported that natural agarwood takes 20 years onward to be produced, and Sadgopal [9], as cited in [7], suggested that the best yields of resin are produced from trees aged 50 years and older. However, not all *Aquilaria* trees produce agarwood, and it was estimated that only 10% of wild *Aquilaria* spp. are infected and produce resin [10]. Due to this, it is difficult to meet the increasing demand for agarwood.

To prevent the extinction of *Aquilaria* trees and meet demand, some countries have taken the initiative to make *Aquilaria* plantations and artificially induce the production of agarwood in the trees. In Vietnam, a nongovernmental organization known as The Rainforest Project (TRP) developed field experiments for the artificial induction of agarwood. The experiment was performed by deliberately wounding the trees by drilling the trees and keeping the wound open with a small piece of plastic pipe, and injecting chemicals to stimulate the trees’ defense mechanism to produce resin. This artificial induction was able to yield agarwood 10 times faster than natural formation and has been considered ‘one of the most successful findings’ [4]. This is supported by a recent study by Mohammad et al. [11], in which *A. beccariana* and *A. microcarpa* in Brunei Darussalam were inoculated with fungi isolated from various locations in the country and were able to produce agarwood within a month.

This review will analyze in-depth the different induction approaches (physical, chemical, and biological) and compare whether the production, yield, and quality of artificially induced agarwood varies from naturally formed agarwood. This will provide insight into how to prevent the overexploitation of *Aquilaria* trees in the wild while trying to meet the increasing demand for agarwood in the world. Additionally, fungal interactions with *Aquilaria* trees will also be explored in this paper to provide a better understanding of why resin is produced when the trees are stressed.

## 2. Natural Agarwood

Agarwood is formed when *Aquilaria* trees are wounded and exposed to biotic and abiotic stresses [12]. The infection triggers the trees’ defense mechanism, causing resin to be produced, which aids the trees in suppressing the growth of the microbes infecting the trees in a process known as tylosis [7,12]. From the infection, the tree undergoes a biochemical reaction that produces oleoresin, which causes the color of the wood to eventually change from a lighter to a darker color, becoming what is commonly referred to as agarwood [12]. Wild-type agarwood takes years to produce, and few traders are willing to wait so long. Furthermore, only a small number of *Aquilaria* are infected in the wild and produce agarwood, and the only way to be certain that the tree contains the desired resin is to cut down the trees [4].

## 3. Artificially Induced Agarwood

Methods of artificial induction of agarwood have been created to prevent *Aquilaria* trees from becoming extinct. This causes the trees to become endangered, and, therefore, researchers have produced methods to artificially induce agarwood formation. There are three methods used: biological inoculation, chemical induction, and physical wounding. Table 1 summarizes the different induction mechanisms involved in the agarwood formation along with the details of its quality.

### 3.1. Physical Wounding

Mechanical injuries are the common and traditional method used to induce agarwood formation, as it is cheap and is inexpensive, requiring no chemicals or reagents to be used. It is also much easier to teach methods of mechanical injuries to farmers who cultivate agarwood. In China, farmers in Hainan, Guangdong, and Yunnan provinces were taught the physical wounding method to cultivate more than 20 million *A. sinensis* trees [15]. Ponjanagroon and Kaewrak [13] have used various methods of mechanical injuries on *A. crassna* to induce the production of agarwood; they inflicted wide and narrow wounds on the trees, made holes on the trees with screws of varied sizes, severe bark removal with hatchets, inserted nails of assorted sizes into the tree trunk, and the last one is to simply beat the *Aquilaria* trunk with a hammer. All methods produced discoloration; however, when the wood is burnt, the wood with nails hammered into the trunk gave no specific agarwood scent. Nobushi and Siripatanadilok [20] suggested that air and oxygen play a role in agarwood formation. Thus, when the nails are hammered into the trunk of the trees, oxygen is not able to enter the wound, and the discoloration around the wound could be caused by the reaction of ferric oxide in the nails and wood fibers [13]. Hence, there is no aromatic scent when burning the wood, as little or no resin was formed. The study also concluded that larger objects used to injure the trees cause wider discoloration, and the holes wounded with large screws were preferred, as it produces the classic agarwood scent when burnt and the quantity of agarwood at 20 months (about 1 and a half years)’ harvest was still not enough for commercial purposes [13].

### 3.2. Biological Inoculation

Biological inoculation is also another alternative method to agarwood formation and has been proven by many researchers to help induce agarwood formation. It is necessary for the tree to first be wounded before it can be infected by microbes to induce agarwood formation. However, not all fungi can promote agarwood production; some of the species identified in agarwood-producing trees are *Fusarium*, *Lasiodiplodia*, *Penicillium*, and *Aspergillus*, amongst others [12]. Inoculation of endophytic fungi on *Aquilaria* trees has also been proven to produce resin in as fast as 6 months [21]. Chen et al. [22] studied the agarwood formation induced by fermentation liquid of different fungi, in which the fungi were isolated from a previously infected tree that produced agarwood and were inserted into the *Aquilaria* tree by using a transfusion set. It was found that the dominant fungi were *Lasiodiplodia theobromae*, which was present in all layers of the wood, followed by *Fusarium solani* [22]. This suggests that *L. theobromae* and *F. solani* have a significant role in agarwood production and are agarwood-promoting fungi.

### 3.3. Chemical Induction

Chemical induction is another common method of producing agarwood in many countries. It is common to use sulfuric acid, jasmonic acid, acetic acid, and alcohol to induce agarwood formation, of which jasmonic acid has been proven to induce agarwood formation by 2–3 mm (about 0.12 in) thickness in Vietnam [17]. However, some countries have used sulfuric acid and acetic acid with unsuccessful results, and some chemicals are toxic to humans, hence the importance of choosing the proper chemicals when the agarwood is intended to be used for making perfumes, tea, and medicines [17]. Methods for injecting the chemicals in agarwood are similar in many reports, in which a hole is drilled into the trunk of the tree, and the chemicals are injected into the tree using a syringe or transfusion set [15,17,18]). There are kits and techniques made by researchers to induce agarwood production, such as the cultivated agarwood kits (CA-Kits) developed by Prof. Blanchette from the University of Minnesota, Vietnam, the whole-tree agarwood-inducing technique (Agar-Wit) that was developed in China [15]), and the agarwood inducement method (AINM) developed by Nuclear Malaysia, in which small holes of about 50 cm are drilled into the xylem of a tree followed by injection of agarwood inducers into the xylem. The resin can then be harvested after 6 months [18].

## 4. Biological Induction and Biosynthesis of Resin

Many endophytic fungal species have been reported to play a vital role in agarwood resin production in the *Aquilaria* and *Gyrinops* species [23]. Table 2 describes the various fungal endophytes known to induce agarwood formation in the *Aquilaria* and *Gyrinops* species. These fungal species could be inoculated into the trees either by natural (naturally occurring endophytes) or through artificial inoculation (through artificially created wounds or openings) methods and as pure or mixed cultures enabling the stimulation of the plants’ immune response favoring resin production [24,25,26,27]. Biological induction is always considered the efficient method in resin formation, as it is safer, healthier, and ecofriendly compared with other methods and is a continuous process compared with the natural and physical induction methods [17]. Various sequential processes catalyzed by a set of specific enzymes in the plant cells enable the formation of the resinous materials, namely chromones and sesquiterpenoids in the plants.

The 2-(2-phenylethyl) chromones (PECs) and sesquiterpenoids are the major secondary metabolites synthesized by the trees along with triterpenes and sterols due to the stress developed by the natural and artificial induction methods on the immune system [58]. Chromones with a remarkably high and wide range of medicinal and therapeutic values [59,60,61] are derived from benzopyrans (polycyclic organic compound) with a keto group in the oxime ring. Biosynthesis of the chromones is initiated through multiple mechanisms involving the pentaketide pathway, shikimic acid pathway, and addition of nitrogenous groups from amino acids to the chromones [59,62]. Recent studies by Wang et al. [63] have shown that the stress caused by elevated levels of salinity has improved resin production in the *Aquilaria* tress. Similarly, according to Liao et al. [64], flindersia-type 2-(2-phenylethyl) chromones, an important and major constituent of the resin, were found to be formed by the catalysis of III polyketide synthase (PKs) through condensation of dihydro-cinnamoyl-CoA analogs and malonyl-CoA with 2-hydroxy-benzoyl-CoA, and on subsequent catalysis with hydroxylases or O-methyltransferases (OMTs). As PECs are synthesized using a complex phenomenon involving a sequence of processes in the plants, it is still a challenging task for researchers worldwide to elucidate the exact pathway involved in the synthesis of PECs. Researchers Goel and Makrandi [65] and Tawfik et al. [61] have stated that the known pathways of PECs synthesis to date were found to be incomplete, with inadequate information lacking the proper understanding of the specific linkages in the pathway of the PECs synthesis. Conversely, sesquiterpenes, triterpenes, and sterols are synthesized using the isoprenoid precursor with the mevalonic acid pathway in the cytosol of the plant cells, and the methylerythritol phosphate pathway acts as a precursor that helps in the synthesis of monoterpenes, diterpenes, and carotenoids in the plastids [66,67,68]. Both the pathways discussed above initiate the synthesis of C5 homoallylic isoprenoid precursors such as isopentenyl pyrophosphate and dimethylallyl pyrophosphate (which are found to be exchanged among the two pathways in the space between the cytosol and plastids), engaging pyruvate, acetyl-CoA, and various other enzymes [67]. These C5 homoallylic isoprenoid precursors on sequential condensation in the presence of C15 farnesyl pyrophosphate synthase generates C15 farnesyl pyrophosphate [66,69]. Researchers such as Yang et al. [69] and Liu et al. [70] have stated that C15 farnesyl pyrophosphate synthase acts as the key limiting factor in the synthesis of sesquiterpenes that are encoded by the gene *Am-FaPS-_1_* and *AsFPS_1_* in *Aquilaria* species. Another set of enzymes, namely sesquiterpene synthase and cytochrome P450 dependent mono-oxygenases, play a pervasive role in the final stage of the sesquiterpene synthesis by oxidative functionalization of the C15 farnesyl pyrophosphate with the cytochrome P450 dependent mono-oxygenases, thus forming a multicyclic scaffold complex. These scaffolds are further modified by the addition of functional groups by alkylation, esterification, and the addition of sugar residues to the hydroxyl end generated by the cytochrome P450-dependent mono-oxygenases in the scaffolds, thus forming different known sesquiterpenoids [71,72]. Further, many studies are underway to better understand the synthesis pathway of the sesquiterpenoids and the role of different genes involved in their synthesis and the synthesis of various synthases involved in the process.

## 5. Agarwood Quality

The market value of agarwood is determined by its quality. Agarwood quality grading is often supervised by trained human graders who base the quality on color, odor, high fixative, and consumer perception [2]. Different countries have their own ways of labeling agarwood quality; some countries prefer to use the terms ‘high quality’ and ‘low quality,’ and other countries prefer to categorize agarwood into groups A, B, C, or D [73]. However, it is subjective when agarwood quality is determined by humans, in which each person might have different opinions on the physical appearance of agarwood. In addition, humans are prone to fatigue and nausea when exposed to many fragrances for a prolonged period, and this would limit the analysis and may cause wrong judgment to be made. The agarwood compound is associated with the presence of sesquiterpenes, which are the main active compounds in agarwood that gives it the fragrant odor [74]. Thus, the more sesquiterpenes the agarwood contains, the higher the quality of the oil would be.

The three methods of artificial induction come with their own advantages and disadvantages. The physical-mechanical method is by far the most common method and is also known as the traditional method. According to researchers [13,16], this method produces low-grade and low-yield agarwood and often takes a long time to produce agarwood. However, this method is common, as it is easy to teach farmers who cultivate agarwood and is cheap cost-wise. As for the biological method, the quality and yield depend on the strains of fungi or bacteria used during the inoculation process. Some studies have mentioned *Aspergillus* sp., *L. theobromae*, and *Fusarium* sp. [12,22] as playing a significant role in the biological inoculation of agarwood. The quality of agarwood is then determined by analyzing the sesquiterpenes compounds that are present. As for the production time, agarwood can be produced in as early as 6 months [21], but the yield of agarwood from this method needs to be further evaluated when using different strains.

In terms of chemical induction, Chong et al. [18] harvested agarwood from trees that were 18 months (about 1 and a half years) old, in which some of the trees were inoculated with fungi, and the others were injected with chemical inducers using AINM. Their results showed that the yield of agarwood using chemical inducers was 12.9 times higher than using fungal strains [18]. Another study by Liu et al. [15] also showed that agarwood collected from the Agar-Wit method after 6 months was also superior compared with physical wounding and biological inoculation. High-quality agarwood had a high alcohol soluble extractive content, and the samples collected using the Agar-Wit method had an alcohol soluble content surpassing the 10% standard, which was similar to the agarwood obtained from wild *Aquilaria* trees [15]. Furthermore, when the harvest occurred after 12 or 20 months (about 1 and a half years), the agarwood produced was an even higher quality [15]. Additionally, in these studies, the quality of agarwood oil and the yield using chemical inducers compared with other methods had high levels of the sesquiterpenes compound, which shows that chemical induction is the best method to produce agarwood that has similar qualities to wild agarwood.

## 6. Bioactive Compounds Obtained from Agarwood, Their Pharmaceutical Properties, and Medicinal Benefits

Agarwood and its products, either as oil, smoke, or powder admixtures, are well known for their bioactivity in controlling various fungal pathogens and their unique medicinal properties globally [75]. Several chemical compounds have been reported to be identified from agarwood such as chromone derivates, terpenoids, flavonoids, benzophenones, lignans, benzenoid derivates, phenolic compounds, triterpenes, steroids, and other chemical compounds [58,76]. Of these, chromones and terpenoids (sesquiterpenoids) are the compounds of interest that are potentially known for their bioactivity, pharmaceutical value, and medicinal properties [76]. In contrast, the other chemical compounds are natural compounds observed in most plants and trees [77].

### 6.1. Chromones

More than 80 different chromones (2-(2-phenylethyl) chromones) and about 31 different terpenoids are known to date (Table 3) that are responsible for various medicinal benefits [78]. Chromones (1,4-benzopyrone) are the known isomer of coumarin, which are derivatives of benzopyran with a substituted keto group in the pyran ring, are ubiquitously present in the Pant Kingdom, and are a part of the human and animal diet [79]. The 2-(2-phenylethyl) chromones are the rare and uncommon group of chromones that possess phenylethylene at the C-2 position, which are reported to be abundantly available in agarwood resin (Table 3) [80,81]. These 2-(2-phenylethyl) chromones have been isolated only from a few plant species, making it a rare compound extracted from plants such as agarwood that are responsible for the warm, balsamic, long-lastingly sweet odor of the burnt agarwood [82]. Furthermore, they are classified into four subgroups, namely 2-(2-Phenylethyl)chromone monomers (PEC), comprising four subdivisions (Flindersia-type 2-(2-phenylethyl)chromones, the most abundant group; 5,6,7,8-Tetrahydro-2-(2-phenylethyl)chromones, highly oxidizing group; mono-epoxy-5,6,7,8-tetrahydro-2-(2-phenylethyl)chromones; diepoxy-5,6,7,8-tetrahydro-2-(2-henylethyl)chromones), 2-(2-Phenylethenyl)chromones are predominantly obtained from chemical synthesis, agarwood, cyanobacteria, and rhizomes of *Imperata cylindrica* or *Platanus x acerifolia*; dimeric 2-(2-phenylethyl)chromones; sesquiterpenoid-4H-chromones and benzylacetone-4H-chromones, predominantly obtained from *Gyrinops salicifolia*; and trimeric 2-(2-phenethyl)chromones [64,83,84,85,86,87]. They act as potential and remarkable pharmacological compounds containing various bioactivities such as antimicrobial, antiviral, anticancer, antitumor, anti-inflammatory, antioxidant, enzyme inhibition, antifeedant, antidepressant, antiobesity, and antihypersensitive properties, including antagonistic activity in melanin-concentrating hormone receptor-1 [88,89,90].

Furthermore, chromones are considered naturally available products with diverse structures and functions that act as potential candidates for replacing synthetic drugs in various pharmacological therapeutics [89,113,114,115,116,117,118,119,120,121,122,123,124,125,126]. Similarly, Duan et al. [127] and Vanguru et al. [128] have reported the therapeutic properties of chromones against a wide range of cancers, especially in controlling breast cancer and ovarian cancer [129,130]. They act as intercellular adhesion molecule inhibitors, cyclooxygenase inhibitors, mast cell stabilizers, leukotriene receptor antagonists, interleukin-5 inhibitors, lipoxygenase inhibitors, and nitric oxide production inhibitors controlling inflammation as potential anti-inflammatory compounds [92,131,132,133,134,135,136,137,138,139,140,141,142,143,144,145,146]. Chromones derived from the leaf extracts of plants act as the basic structural compound in the development of various drugs that inhibit infectious diseases caused by a wide range of microbes inferring its antimicrobial property [147,148,149,150,151]. Reactive oxygen species (ROS) act as oxygen moieties impairing the DNA, lipids, proteins, and lipoproteins with their oxidative damage [152,153,154]. Chromones present in the food material (plants) act as potential antioxidants in reducing the lipid peroxidase activity of the ROS and free radicals and disease progression [155,156,157,158,159,160,161,162]. In particular, chromones have been found to be promising in controlling and treating the neurodegenerative disease Alzheimer’s, which is caused due to the redox imbalance created by the free radicals and the ROS in the human brain [62,163]. Valentina et al. [164] and Wang et al. [91] have investigated and reported the inhibitory effect of the chromones on the α-amylase and α-glucosidase enzymes to manage postprandial diabetes by delaying sugar uptake in the human body. Further, the role of chromones in the treatment of various disorders such as gastritis, diarrhea, stiff muscles, hypothermic disease, diuretic disease, nephritis, cystitis, pyrexia, rheumatism, headache, hepatitis, cough, bronchitis, asthma, etc. have been reported and described by various researchers worldwide (Table 3) [88,89,90].

### 6.2. Terpenoids

Terpenoids are abundantly present in nature, especially in plants, and are known to contain oxygen derivatives that bear a hydroxyl group at the C-3 position [77]. To date, about three different terpenoids have been identified in agarwood and *Aquilaria* plant leaves, namely 3-oxo-22-hydroxyhopane, 3b-olean-12-ene-3,23-diol, and hederagenin [13,70,97]. Among all the terpenoids known, sesquiterpenoids are the dominant fragrant compounds observed to be present in agarwood and agarwood products with the presence of three isoprene structural units (Table 3) [82,165]. They exist as volatile compounds in the essential oils extracted from agarwood and are of several types with unique aromatic properties specific to each type [77]. Eudesmanes, otherwise known as selinanes, are bicyclic sesquiterpenoids with a decalin skeleton, diverse functional groups, and multiple chiral centers acting as significant sesquiterpenoids found in agarwood obtained from *Aquilaria agallocha, A. sinensis, A. crassna, A. malaccensis,* and *Gyrinops salicifolia* [13,84,86,103,166,167]. They possess a sweet, woody, honey, balsamic, minty, and fresh floral odor [82]. Guaianes are the sesquiterpenoids made of a five- and seven-membered ring-coupled structures containing 4,10-dimethyl-7-isopropenyl moiety and are observed in *Aquilaria* and *Gyrinops* species with a woody and floral odor [84,97,104,168]. Agarospiranes sesquiterpenes, also known as vetispiranes, are composed of spirocyclic sesquiterpenes present in *Aquilaria agallocha, A. sinensis, A. crassna,* and *A. malaccensis* with a spicy, peppery, woody, sweet, and balsamic odor [82,168,169,170]. Eremophilanes, known as valencanes, are similar to eudesmanes structurally and differ due to the presence of a specific methyl group in the structure with a characteristic warm woody and minty odor [13,84,86]. Acoranes, responsible for the long-lasting odor of agarwood, are the spiro sesquiterpenes that are rarely obtained from agarwood [97,103,171]. Cadinanes, a bicyclic sesquiterpene, is similar to eudesmanes with the presence of an isopropyl and methyl group in its structure obtained from *A. sinensis* and *A. crassna* [13,172]. Prezizaanes are the tricyclic sesquiterpenes found in *A. malaccensis* with a special fragrance [171,173,174]. Similarly, Zizaanes, a tricyclic, and Humulanes, a humulane-type sesquiterpenes, have also been reported to be obtained from *A. sinensis and A. crassna* [103,171,175]. Further, other sesquiterpenoids such as daphnauranols obtained from *A. malaccensis*, 12-Hydroxy-dihydrocyperolone from *G. salicifolia*, malacinones A and B from *A. malaccensis*, and 1,5,9-trimethyl-1,5,9-cyclododecatriene from *A. sinensis* have been reported by researchers such as Ma et al. [175], Li et al. [97], Chen et al. [176], and Ma et al. [177]. Among all the sesquiterpenoids reported, eudesmanes, eremophilanes, and guaianes are the potential sesquiterpenoids obtained from agarwood of which guaianes are the compounds possessing high structural diversity that are specific to each plant species [77].

Furthermore, sesquiterpenes act as a potential neuroprotective agent and help in combating Alzheimer’s disease [178]. Similarly, agarol obtained from *A. malaccensis*, an eudesmane sesquiterpene, and 8bH-Dihydrogmelofuran and gmelofuran, a cadinene sesquiterpenes isolated from *A. malaccensis* and *A. agallocha*, were reported to have anticancer, antioxidant and antimicrobial properties [179]. Antidiabetes activity has been observed in some sesquiterpenoids such as Prezizaane, jinkohol II, aquilarene D, jinkohol, zizaane, agarozizanol E, and isokhusenol acting as inhibitors against α-glucosidase [180].

Moreover, cucurbitacin triterpenoids were found to possess cytotoxic activities, making them a potential candidate for the treatment of cancer [181]. Similarly, β-Caryophyllene, isolated from *A. crassna*, was found to specifically help in the de-proliferation of cancerous cells. Further, β-Caryophyllene was a potential antimicrobial compound effective against various pathogenic strains that include *Bacillus cereus, B. subtilis, S. aureus, Escherichia coli, Klebsiella pneumoniae, Pseudomonas aeruginosa, Aspergillus niger, Penicillium citrinum, Rhizopus oryzae,* and *Trichoderma reesei* [179]. Flavonoids, benzophenones, xanthones, lignans, phenolic compounds, degraded sesquiterpenes, alkaloids, and nucleosides are some of the other chemical constituents of the *Aquilaria* species that are available compounds in plants, and they contribute to the structural stability of the plants [58].

## 7. Economic Importance and Market Value of Agarwood

Aquilaria trees and the agarwood obtained from them are very popular for the wide variety of economically important products obtained from them [182]. Their products, such as wood, wood chips, oil, powder, and flakes, have been used in various applications such as medicinal, religious, cosmetics, and cultural purposes [183,184]. Antonopoulou et al. [185], Barden et al. [1], Kiet [186], Lim and Anack [187], Persoon [188], and Sitepu et al. [189] have reported the potential usage of agarwood oil and powder in Ayurvedic medicine practiced in the Indian subcontinent, and also in Tibetan, Vietnamese, Chinese, and Malaysian medicines. Moreover, it is used for religious and cultural purposes in many countries such as Northeast and Southeast Asia, Taiwan, Korea, and Japan [184,185,190]. Furthermore, Sitepu et al. [189], Barden et al. [1], and Chakrabarty et al. [8] have reported the importance of agarwood in the manufacturing of perfumes, cosmetic products, soaps, shampoos, incense, and other fragrance products worldwide.

Therefore, the market value of agarwood and the demand for its products have increased tremendously. Globally, agarwood products, such as wood pieces, wood chips, powder, oil, dust, incense, and perfumes, have been reported to have a market value of USD several thousand billion [1,182,191]. Agarwood is graded into different categories based on the resin quality; the first grade has a high global demand increasing the market value to about USD 10,000 per kg of wood, and the least grade with USD 30 per kg [192]. Further, according to a report by Nanyang Siang Pau in 2005 and Abdin [193], agarwood oil is generally sold at a cost of USD 30,000 to 40,000 per kg. Similarly, in 2013, Akter et al. [7] reported an estimated increase in the market value of agarwood and its products to reach up to USD 6–8 billion or even up to USD 36 billion by 2017. Similarly, other reports from various sources indicate that the agarwood oil market value is expected to reach about USD 201.03 million by 2026. Hence, it **is** essential to better understand the basic scientific concepts of agarwood induction and formation to improve its production. Furthermore, the invention of simple technology for induction would enable the easy handling of the techniques by farmers to induce the crops.

## 8. Conclusions

Agarwood formation in the natural environment, although mysterious, can be produced through artificial inoculation techniques. Agarwood is highly sought after due to its economic value and cultural and medicinal uses around the world. Its high value has caused indiscriminate logging of wild *Aquilaria* trees, thereby causing dominant species to become critically endangered. Therefore, methods of artificial induction are developed to prevent its extinction and reduce the burden on wild *Aquilaria*. The quality of artificially induced agarwood is close to wild agarwood, and its production in *Aquilaria* plantations may be able to meet the ever-increasing demand for this unique fragrance. Several applications, including perfumery and therapeutics, further substantiate the ongoing investigations into agarwood and the growing impetus for sustainable cultivation.

## Figures and Tables

**Table 1:** Types of wounds inflicted on *Aquilaria* trees and the method to inflict these wounds for agarwood production. The quality and yield of agarwood are dependent on the method of wound infliction.

Wounding Type	Method	Quality and Yield	References
Natural	Lightning strike, animal grazing, insect attack, microbial invasion on wounded parts	High quality and low yield	[13,14]
Biological	Bacterial and fungal strains cultured on agar and injected into *Aquilaria* trees	Quality and yield depend on the strain used for the inoculation—can be either high or low quality and yield	[10,15,16]
Chemical	Whole-tree agarwood-inducing technique (Agar-wit), cultivated agarwood kit (CA-Kits), agarwood inducement method (AINM), jasmonic acid, acetic acid, sulfuric acid, alcohol, phytohormones, salts, minerals, biologically derived substances	Similar quality to natural agarwood and more yield compared with biological and physical wounding; however, both yield and quality depend on the chemicals used	[15,16,17,18,19]
Physical	Axe wounds, severe bark removal, nailing on tree trunk, partial trunk pruning method, burning-chisel-drilling method	Low quality and low yield	[1,13,16]

**Table 2:** Resin-inducing endophytic fungal species isolated from the *Aquilaria* and *Gyrinops* species.

Fungal Endophyte	Specific Plant Species Reported for Resin Production	References
*Aspergillus* sp.	*Aquilaria* sp.	[28,29,30]
*Botryodyplodis* sp.		
*Botryosphaeria dothidea*		
*Diplodia* sp.		
*Fusarium bulbigenium*		
*Fusarium lateritium*		
*Fusarium oxysporum*		
*Fusarium* sp.		
*Fusarium moniliforme*		
*Fusarium sambucinum*		
*Fusarium solani*		
*Fusarium tricinctum*		
*Epicoccum granulatum*	*Aquilaria agallocha*	[9,31,32,33,34]
*Cladosporium* sp.		
*Torula* asp.		
*Chaetomium globosum*		
*Fusarium oxysporum*		
*Melanotus flavolivens*	*Aquilaria sinensis*	[35,36,37,38,39,40,41,42,43,44,45,46]
*Lasiodiplodia* sp.		
*Xylaria* sp.		
*Paraconiothyrium variabile*		
*Botryosphaeria* sp.		
*Fusarium* sp.		
*Lasiodiplodia theobromae*		
*Fusarium oxysporum*		
*Rigidoporus vinctus*		
*Nigrospora oryzae*		
*Fusarium solani*		
*Acremonium* sp.	*Aquilaria microcarpa*	[47,48]
*Fusarium* sp.		
Unidentified *Deuteromycetes*	*Aquilaria malaccensis*	[49,50,51,52,53]
Unidentified *Ascomycetes*		
*Fusarium* sp.		
*Fusarium solani*		
*Acremonium* sp.	*Aquilaria crassna*	[54]
*Fusarium* sp.		
*Fusarium solani*		
*Lasiodiplodia theobromae*		
*Fusarium* sp.	*Aquilaria beccariana*	[55]
*Fusarium* sp.	*Gyrinops versteegii*	[30,53,56]
*Fusarium solani*		
*Aspergillus niger*	*Gyrinops walla*	[57]
*Fusarium solani*		

**Table 3:** List of chromones and terpenoids known to be extracted from agarwood obtained from **Aquilaria sinensis*, A. malaccensis, A. filaria*, and *Gyrinops versteegii* (as reported by Wang et al. [91] and Yuan et al. [76]).

Chemical Compounds	Bioactive Properties	References
Chromones		
6-hydroxy-7-methoxy-2-[2-(3′-hydroxy-4′-methoxyphenyl)ethyl]chromone	Antioxidant, analgesic, digestive, tumor cell inhibition, enzyme inhibition, antimicrobial, antiplasmodial, antifeedant, immunomodulatory, anti-inflammatory, antitubercullii Treatment of gastritis, diarrhea, stiff muscles, hypothermic disease, diuretic disease, CNS activity, nephritis, cystitis, urethral disease, pyrexia, rheumatism, headache, hepatitis, cough, bronchitis, asthma	[60,92,93]
6,7-dimethoxy-2-[2-(3′-hydroxy-4′-methoxyphenyl)ethyl]chromone		[60,93]
7-hydroxy-6-methoxy-2-[2-(3′-hydroxy-4′-methoxyphenyl)ethyl]chromone		[60]
6,7- dimethoxy-2-[2-(4′-hydroxy-3′-methoxyphenyl)ethyl]chromone		[60,93]
6,7-dihydroxy- 2-[2-(4′-methoxyphenyl)ethyl]chromone		[60]
6-hydroxy-7-methoxy-2-[2-(4′- hydroxyphenyl)ethyl]chromone		[60]
6,8-dihydroxy-2-[2-(3′-hydroxy-4′- methoxyphenyl)ethyl]chromone		[60,92,93]
6-methoxy-2-[2-(3-hydroxy-4-methoxyphenyl)ethyl]chromone		[80]
5-hydroxy-6-methoxy-2-[2-(4-methoxyphenyl)ethyl]chromone		[80,94]
6-methoxy-2-[2-(4-methoxyphenyl)ethyl]chromone		[80]
6-methoxy-2-[2-(4-hydroxyphenyl)ethyl]chromone		[80,92]
6-methoxy-2-[2-(3-methoxy-4-hydroxyphenyl)ethyl]chromone		[80,95,96]
6,7-dimethoxy-2-[2-phenylethyl]chromone		[80,94,97]
6-hydroxy-2-(2-phenylethyl)chromone		[80,94]
6-hydroxy-2-[2-(4-methoxyphenyl)ethyl]chromone		[80]
6,8-dihydroxy-2-(2- phenylethyl)chromone		[80]
5-hydroxy-6-methoxy-2-[2-(3-hydroxy-4-methoxyphenyl)ethyl]chromone		[80]
5-hydroxy-6-methoxy-2-[2-(4-methoxyphenyl)ethyl]chromone		[80]
6-hydroxy-7-methoxy-2-[2-(4-methoxyphenyl)ethyl]chromone		[97]
6-methoxy-7-hydroxy-2-[2-(4-methoxyphenyl)ethyl]chromone		[97]
6-hydroxy-2-[2-(3-methoxy-4-hydroxyphenyl)ethyl]chromone		[97]
6-hydroxy-2-[2-(3-hydroxy-4-methoxyphenyl)ethyl]chromone		[92,97]
6-hydroxy-2-[2-(3, 4-dimethoxyphenyl)ethyl]chromone		[97]
6,8-dihydroxy-2-[2-(4-methoxyphenyl)ethyl]chromone		[97]
8-chloro-6-hydroxy-2-[2-(3-methoxy-4-hydroxyphenyl)ethyl]chromone		[97]
5-methoxy-6-hydroxy-2-[2-(3-methoxy-4-hydroxyphenyl)ethyl]chromone		[97]
4′,6-dihydroxy-3′,7-dimethoxy-2-(2-phenyl)ethylchromone		[92]
6-methoxy-2-[2-(3-methoxy-4-hydroxyphenyl)ethyl]chromone		[95]
6-hydroxy-2-[2-(4-hydroxyphenyl)ethyl]chromone		[95]
6,8-dihydroxy-2-[2-(3-methoxy-4-hydroxyphenyl)ethyl]chromone		[96]
7-hydroxy-6-methoxy-2-(2-phenylethyl)chromone		[95]
6,7-dimethoxy-2-(2-phenylethyl)chromone		[95]
6-hydroxy-2-[2-(2-hydroxyphenyl)ethyl]chromone		[98]
7-hydroxy-2-(2-phenylethyl)chromone		[98]
flindersiachromone		[94]
6-methoxy-2-(2-phenylethyl)chromone		[94]
5-hydroxy-6,7-dimethoxy-2-[2-(4′-methoxyphenyl)ethyl]chromone		[81]
8-chloro-6-hydroxy-2-(2-phenylethyl)chromen-4-one		[99]
8-chloro-6-hydroxy-2-[2-(4-methoxyphenyl)-ethyl]chromen-4-one		[99]
aquilarinoside C		[100]
6-hydroxy-2-[2-(40-hydroxy-30-methoxyphenyl)ethenyl]chromone		[60]
(5S,6S,7S,8R)-2-[2-(3-hydroxy-4-methoxyphenyl)ethyl]-5,6,7,8-tetrahydroxy-5,6,7,8-tetrahydrochromone		[92]
(5S,6S,7S,8R)-2-(2-phenylethyl)-5,6,7,8-tetrahydroxy-5,6,7,8-tetrahydrochromone		[92]
(5S,6S,7S,8R)-2-[2-(4-methoxyphenyl)ethyl]-5,6,7,8-tetrahydroxy-5,6,7,8-tetrahydrochromone		[92]
(5S,6R,7S,8R)-2-[2-(3-hydroxy-4-methoxyphenyl)ethyl]-5,6,7,8-tetrahydroxy-5,6,7,8-tetrahydrochromone		[92]
(5S,6R,7R,8S)-2-[2-(3-hydroxy-4-methoxyphenyl)ethyl]-5,6,7,8-tetrahydroxy-5,6,7,8-tetrahydrochromone		[92]
(5S,6R,7R,8S)-2-[2-(4-hydroxyphenyl)ethyl]-5,6,7,8-tetrahydroxy-5,6,7,8-tetrahydrochromone		[92]
8-chloro-2-(2-phenylethyl)-5,6,7-trihydroxy-5,6,7,8-tetrahydrochromone		[92]
6S,7R)-5,6,7,8-Tetrahydro-6,7-dihydroxy-2-(2-phenylethyl)-4H-1-benzopyran-4-one		[94]
aquilarone F		[94]
(5R,6R,7R,8S)-8-chloro-5,6,7-trihydroxy-2-(4-methoxyphenethyl)-5,6,7,8-tetrahydrochromone		[81]
(5S,6S,7S,8S)-8-chloro-5,6,7-trihydroxy-2-(2-phenylethyl)-5,6,7,8-tetrahydrochromone		[81]
(5R,6R,7R,8R)-8-chloro-5,6,7-trihydroxy-2-(4-methoxyphenethyl)-5,6,7,8-tetrahydrochromone		[81]
(5R,6S,7S)-5,6,7-trihydroxy-2-(4-hydroxy-3-methoxyphenethyl)-5,6,7,8-tetrahydrochromone		[81]
tetrahydrochromone A		[101]
tetrahydrochromone B		[101]
tetrahydrochromone C		[101]
tetrahydrochromone D		[101]
tetrahydrochromone E		[101]
tetrahydrochromone F		[101]
tetrahydrochromone G		[101]
tetrahydrochromone H		[101]
tetrahydrochromone I		[101]
tetrahydrochromone J		[101]
(5R,6R,7S,8R)-5,6,7,8-tetrahydroxy-2-(2-phenylethyl)-5,6,7,8-tetrahydrochromone		[84]
agarotetrol		[84]
qinanmer		[84]
5,6-epoxy-7β-hydroxy-8β-methoxy-2-(2-phenylethyl)chromone		[80]
rel-(1aR,2R,3R,7bS)-1a,2,3,7b-tetrahydro-2,3-dihydroxy-5-(2-phenylethyl)-7H-oxireno-benzopyran-7-one		[80]
rel-(1aR,2R,3R,7bS)-1a,2,3,7b-tetrahydro-2,3-dihydroxy-5-[2-(4-methoxyphenyl)ethyl]-7H-oxireno-ben zopyran-7-one		[94]
oxidoagarochromone A		[80]
tetrahydrochromone L		[101]
tetrahydrochromone M		[101]
oxidoagarochromone A		[80]
oxidoagarochromone B		[80]
oxidoagarochromone C		[101]
(R)-6,7-dimethoxy-2-(2-hydroxy-2-phenylethyl)chromone		[93]
(S)-6,7-dimethoxy-2-(2-hydroxy-2-phenylethyl)chromone		[93]
AH10		[94]
AH14		[94]
(5S,6R,7S,8R)-2-[2-(4-Methoxyphenyl)ethyl]-5,6,7-trihydroxy-5,6,7,8-tetrahydro-8-{6- methoxy-2-[2-(3‴-methoxy-4‴-hydroxyphenyl)ethyl]chromonyl-7-oxy}chromone		[102]
(5S,6R,7S,8R)-2-[2-(4-Methoxyphenyl)ethyl]-5,6,7-trihydroxy-5,6,7,8-tetrahydro-8-{2-[2-(4‴-methoxy phenyl)ethyl]chromonyl-6-oxy}chromone		[102]
(5S,6R,7S,8R)-2-(2-Phenylethyl)-5,6,7-trihydroxy-5,6,7,8-tetrahydro-8-[2-(2-phenylethyl)chromonyl-6- oxy]chromone		[102]
(5R,6R,7R,8S)-2-(2-Phenylethyl)-5,6,7-trihydroxy-5,6,7,8-tetrahydro-8-[2-(2-phenylethyl)chromonyl-6- oxy]chromone		[102]
aquisinenone A		[81]
(−)-4′-methoxyaquisinenone A		[81]
aquisinenone B		[81]
(−)-6″-hydroxyaquisinenone B		[81]
(+)-6″-hydroxy-4′,4‴-dimethoxyaquisinenone B		[81]
aquisinenone C		[81]
(−)-aquisinenone D		[81]
4′-demethoxyaquisinenone D		[81]
(+)-aquisinenone E		[81]
(−)-aquisinenone F		[81]
(−)-aquisinenone G		[81]
(+)-4′-methoxyaquisinenone G		[81]
**Terpenoids**		
(+)-9β-Hydroxyeudesma-4,11(13)-dien-12-al	Antimicrobial, anti-inflammatory, anticancer, antidiabetes, antioxidants Treatment of immunological disorders, neurological disorders, cancer	[103]
(+)-Eudesma-4,11(13)-dien-8α,9β-diol		[103]
(+)-8α-Hydroxyeudesma-3,11(13)-dien-14-al		[103]
(+)-Eudesma-3,11(13)-dien-8α,9β-diol		[103]
(+)-Eudesma-4(14),11(13)-dien-8α,9β-diol		[103]
(4R,5R,7S,9S,10S)-(−)-Eudesma-11(13)-en-4,9-diol		[103]
(+)-9β,10β-Epoxyeremophila-11(13)-en		[103]
(+)-11-Hydroxyvalenc-1(10),8-dien-2-one		[103]
(−)-Eremophila-9-en-8β,11-diol		[103]
1,10-Dioxo-4H-5H-7H-11H-1,10-secoguaia-2(3)-en-12,8-olide		[104]
1-Hydroxy-4H-5H-7H-11H-8,9-secoguaia-9(10)-en-8,12-olide		[104]
1-Hydroxy-4α,10α-dimethyl-5H-octahydro-azulen-8-one		[104]
1α-Hydroxy-4α,10α-dimethyl-5βH-octahydro-azulen-8-one		[104]
4-Hydroxyl-baimuxinol		[105]
7β-H-9(10)-ene-11,12-Epoxy-8-oxoeremophilane		[105]
7α-H-9(10)-ene-11,12-Epoxy-8-oxoeremophilane		[105]
(5S,7S,9S,10S)-(+)-9-Hydroxy-selina-3,11-dien-12-al		[106]
(5S,7S,9S,10S)-(−)-9-Hydroxy-selina-3,11-dien-14-al		[106]
(5S,7S,9S,10S)-(+)-9-Hydroxy-eudesma-3,11(13)-dien-12-methyl ester		[106]
(7S,9S,10S)-(+)-9-Hydroxy-selina-4,11-dien-14-al		[106]
(7S,8S,10S)-(+)-8,12-Dihydroxy-selina-4,11-dien-14-al		[106]
Qinanol A		[107]
Qinanol B		[107]
Qinanol C		[107]
Qinanol D		[107]
Qinanol E		[107]
Qinanol F		[107]
3-oxo-7-Hydroxylholosericin A		[97]
1,5,8,12-Diepoxy-guaia-12-one		[97]
(+)-8β-Hydroxy-longicamphenylone		[60]
11β-Hydroxy-13-isopropyl-dihydrodehydrocostus lactone		[60]
Aquilarabietic acid A		[69]
Aquilarabietic acid B		[69]
Aquilarabietic acid C		[69]
Aquilarabietic acid D		[69]
Aquilarabietic acid E		[69]
Aquilarabietic acid F		[69]
Aquilarabietic acid G		[69]
Aquilarabietic acid H		[69]
Aquilarabietic acid I		[69]
Aquilarabietic acid J		[69]
Aquilarabietic acid K		[69]
Aquilarin B		[108]
Aquilanol A		[109]
Aquilanol B		[109]
Daphnauranol D		[109]
Chamaejasmone E		[109]
Aquilacallane A		[110]
Aquilacallane B		[110]
Aquimavitalin		[111]
12-O-(20E,40E)-6-oxohexa-20, 40-Dienoylphorbol-13-acetate		[112]
12-Deoxy-13-O-acetylphorbol-20-(90Z)-octadecenoate		[112]
12-O-(20E,40E)-60, 70-(erythro)-dihydroxytetradeca-20, 40 -dienoylphorbol-13-acetate		[112]
12-O-(20E,40E)-60, 70-(threo)-dihydroxytetradeca-20, 40-dienoylphorbol-13-acetate		[112]

## Data Availability

The data presented in this study are available on request from the corresponding author.
